# Cullin-RING ubiquitin ligases in salicylic acid-mediated plant immune signaling

**DOI:** 10.3389/fpls.2015.00154

**Published:** 2015-03-13

**Authors:** James J. Furniss, Steven H. Spoel

**Affiliations:** ^1^Institute of Molecular Plant Sciences, School of Biological Sciences, University of EdinburghEdinburgh, UK

**Keywords:** Cullin-RING ligase (CRL), ubiquitin ligase, salicylic acid (SA), NPR1, plant immunity, proteasome, transcription activator, gene expression

## Abstract

Plant immune responses against biotrophic pathogens are regulated by the signaling hormone salicylic acid (SA). SA establishes immunity by regulating a variety of cellular processes, including programmed cell death (PCD) to isolate and kill invading pathogens, and development of systemic acquired resistance (SAR) which provides long-lasting, broad-spectrum resistance throughout the plant. Central to these processes is post-translational modification of SA-regulated signaling proteins by ubiquitination, i.e., the covalent addition of small ubiquitin proteins. Emerging evidence indicates SA-induced protein ubiquitination is largely orchestrated by Cullin-RING ligases (CRLs), which recruit specific substrates for ubiquitination using interchangeable adaptors. Ligation of ubiquitin chains interlinked at lysine 48 leads to substrate degradation by the 26S proteasome. Here we discuss how CRL-mediated degradation of both nucleotide-binding/leucine-rich repeat domain containing immune receptors and SA-induced transcription regulators are critical for functional PCD and SAR responses, respectively. By placing these recent findings in context of knowledge gained in other eukaryotic model species, we highlight potential alternative roles for processive ubiquitination in regulating the activity of SA-mediated immune responses.

## Introduction

Successful plant immune responses depend on the rapid recognition of the invading pathogen and subsequent local and systemic transmission of signals that induce resistance throughout all plant tissues. Pattern recognition receptors that recognize conserved pathogen-associated molecular patterns represent the first line of defense, leading to pattern-triggered immunity (Macho and Zipfel, 2014). To subvert immune responses, adapted pathogens have evolved an arsenal of effector proteins that suppress pattern-triggered immunity. The presence of these effector proteins can be sensed by intracellular nucleotide-binding/leucine-rich repeat domain containing (NLR) immune receptors, resulting in effector-triggered immunity (Jones and Dangl, 2006; van Ooijen et al., 2007). Effector-triggered immunity is characterized by rapid onset of programmed cell death (PCD) at the site of infection, which is thought to isolate and prevent proliferation of the invading pathogen. Following pathogen recognition, development of pattern- and effector-triggered immunity requires the immune signaling hormone salicylic acid (SA). Failure to accumulate SA upon pathogen attack results in severe disease susceptibility and inability to launch NLR receptor-mediated PCD (Delaney et al., 1994; Rairdan and Delaney, 2002). Additionally, SA accumulates in tissues adjacent and distant to the site of infection where it induces systemic acquired resistance (SAR), a long-lasting immune response effective against a broad -spectrum of pathogens (Spoel and Dong, 2012; Fu and Dong, 2013). A major function of SA is to initiate reprogramming of the transcriptome to prioritize immune responses over other cellular functions. Accordingly, SA fine-tunes the activity of a network of SA-responsive transcriptional regulators, the concerted action of which establishes disease resistance (Moore et al., 2011).

Recent work has highlighted an important role for the ubiquitin-mediated proteasome system in regulating many aspects of SA-dependent immunity. In eukaryotic cells, post-translational modification by a single or polymeric chain of ubiquitin modulates protein function and stability (Komander and Rape, 2012). Ubiquitin is a highly conserved, small protein (8.5 kDa) that is covalently attached to a target substrate in a multistep enzymatic pathway. First, a ubiquitin-activating E1 enzyme forms a high-energy thioester linkage to a ubiquitin moiety, which is then passed onto an active-site cysteine residue of a ubiquitin-conjugating E2 enzyme. The E2 enzyme works in physical partnership with an E3 ligase to attach ubiquitin onto a specific lysine (Lys) ε-amino group within the target substrate (Smalle and Vierstra, 2004; Komander and Rape, 2012). Compared to many other eukaryotes, plant genomes encode for disproportionally large numbers of E3 ligases; for example, the *Arabidopsis* genome contains over 1400 different predicted E3 ligase components (Vierstra, 2009), suggesting that protein ubiquitination plays critical roles in plant biology. E3 ligases selectively recruit substrates for ubiquitination and thus provide an important level of specificity to the ubiquitination machinery. E3 ligases can be categorized into different classes based on the presence of a RING, U-box, or HECT domain, leading to distinct ways of binding a partner E2 conjugating enzyme. In addition to single polypeptide E3 ligases, the modular multi-subunit family of Cullin-RING Ligases (CRLs) plays prominent roles in protein ubiquitination. The Cullin subunit of CRLs acts as a scaffold to bring together the RING domain-containing protein and a variable adaptor that recruits the target protein (Santner and Estelle, 2009; Vierstra, 2009; Sadanandom et al., 2012). Emerging evidence suggests that plant immune signaling is predominantly mediated by CRL1 (also known as SCF for SKP1/Cullin1/F-box) and CRL3 [also denoted as BC3B for BTB (Bric-à-brac, Tramtrack, and Broad complex)/Cullin3/BTB], which recruit substrate adaptors that contain F-box motifs or BTB domains, respectively.

Although substrate ubiquitination by E3 ligases can have various functions depending on chain topology and length (Komander and Rape, 2012; Walsh and Sadanandom, 2014), ubiquitin chain linkage via Lys48 signals for degradation of the substrate by the 26S proteasome, a large (2.5 MDa) ATP-dependent chambered protease containing over 30 distinct subunits (Pickart and Cohen, 2004).

Several excellent comprehensive reviews are available on the role of ubiquitination in plant immune signaling in general (Trujillo and Shirasu, 2010; Marino et al., 2012; Duplan and Rivas, 2014). Instead, here we specifically focus on recent advances in understanding the function of ubiquitination in SA-induced immune signaling. How processive ubiquitination and degradation of transcription activators may underpin SA-responsive gene expression in local and systemic immunity will be discussed, as well as how CRLs play an integral part of cellular decisions of life and death upon pathogen recognition.

## Ubiquitin-Mediated Suppression of SA-Responsive Gene Transcription

Genetic screens for SA-insensitive *Arabidopsis* mutants have repeatedly identified *npr1* (*non-expresser of PR genes*) mutant alleles (Cao et al., 1994; Delaney et al., 1995; Glazebrook et al., 1996; Shah et al., 1997). *NPR1* encodes a transcription coactivator that in resting cells forms a high molecular weight oligomer in the cytoplasm through intermolecular disulfide bonds between conserved cysteine residues, preventing it from entering the nucleus. Pathogen-induced SA accumulation triggers transient cellular redox changes, resulting in reduction of these disulfide bonds, and release of NPR1 monomers (Mou et al., 2003; Tada et al., 2008). NPR1 monomer translocates to the nucleus where it controls the expression of over 2,200 genes in *Arabidopsis* (Kinkema et al., 2000; Wang et al., 2006), in part by physically interacting with and transactivating TGA transcription factors that associate with SA-responsive gene promoters (Zhang et al., 1999; Després et al., 2000; Zhou et al., 2000; Boyle et al., 2009). NPR1 protein contains an N-terminal BTB domain and a C-terminal ankyrin repeat domain (Cao et al., 1997; Ryals et al., 1997; Aravind and Koonin, 1999). Interestingly, the presence of these domains in a single protein is a typical feature of a substrate adaptor for CRL3, in which the BTB domain mediates interaction with Cullin 3, while the ankyrin repeat recruits substrates for ubiquitination (Petroski and Deshaies, 2005). However, yeast two-hybrid studies were unable to find direct physical interaction between Cullin 3 and NPR1 (Dieterle et al., 2005). Co-immunoprecipitation experiments nevertheless showed that NPR1 associates with a CRL3 *in planta* (Spoel et al., 2009). These results suggested that NPR1 may not be in the substrate adaptor position of this E3 ligase. Indeed, in *Arabidopsis* cells, monomeric NPR1 is itself subject to ubiquitination by a CRL3 and is subsequently degraded in the nucleus. Blocking NPR1 degradation pharmacologically with proteasome inhibitors or genetically by mutation of Cullin 3 resulted in accumulation of NPR1 monomer, moderate induction of NPR1 target genes, and elevated resistance to pathogen infection (Spoel et al., 2009). This indicated that constitutive degradation of NPR1 monomer by CRL3 prevents autoimmunity in absence of a pathogen threat. This suppressive effect of CRL3 and the proteasome probably impacts a large proportion of the immune transcriptome, as many genes are co-regulated by SA and proteasome inhibitor (Spoel et al., 2010).

Ubiquitin-mediated protein degradation plays a similar role in SA-dependent immune responses in rice. Analogous to the function of *Arabidopsis* NPR1, *Oryza sativa* WRKY45 is an SA-induced transcription activator of several hundred immune-related genes and confers resistance to bacterial and fungal pathogens (Shimono et al., 2007, 2012; Nakayama et al., 2013). Inhibition of the proteasome resulted in accumulation of polyubiquitinated OsWRKY45 in the nucleus and constitutive activation of its target genes in the absence of SA treatment (Matsushita et al., 2013). Although it remains unknown if OsWRKY45 is targeted for degradation by a CRL3, these findings indicate that constitutive turnover of this immune activator prevents autoimmune responses. SA also activates an NPR1-like protein, which functions in parallel with OsWRKY45 to regulate immune transcription in rice. By contrast to OsWRKY45, this OsNPR1 protein (also known as OsNH1) is thought to be predominantly involved in downregulation of gene expression, particularly those involved in photosynthetic activity (Sugano et al., 2010). Interestingly, OsNPR1 is not subject to constitutive proteasome-mediated degradation, intuitively suggesting that transcriptional repression does not require corepressor turnover. Hence, the presence of analogous proteasome-regulated modules consisting of unrelated transcription (co)activators in *Arabidopsis* and rice (i.e., NPR1 versus OsWRKY45) may reflect inherent constraints on how timely activation of SA-responsive immune genes can be achieved.

## Ubiquitin-Mediated Activation of SA-Responsive Gene Transcription

**FIGURE 1:**
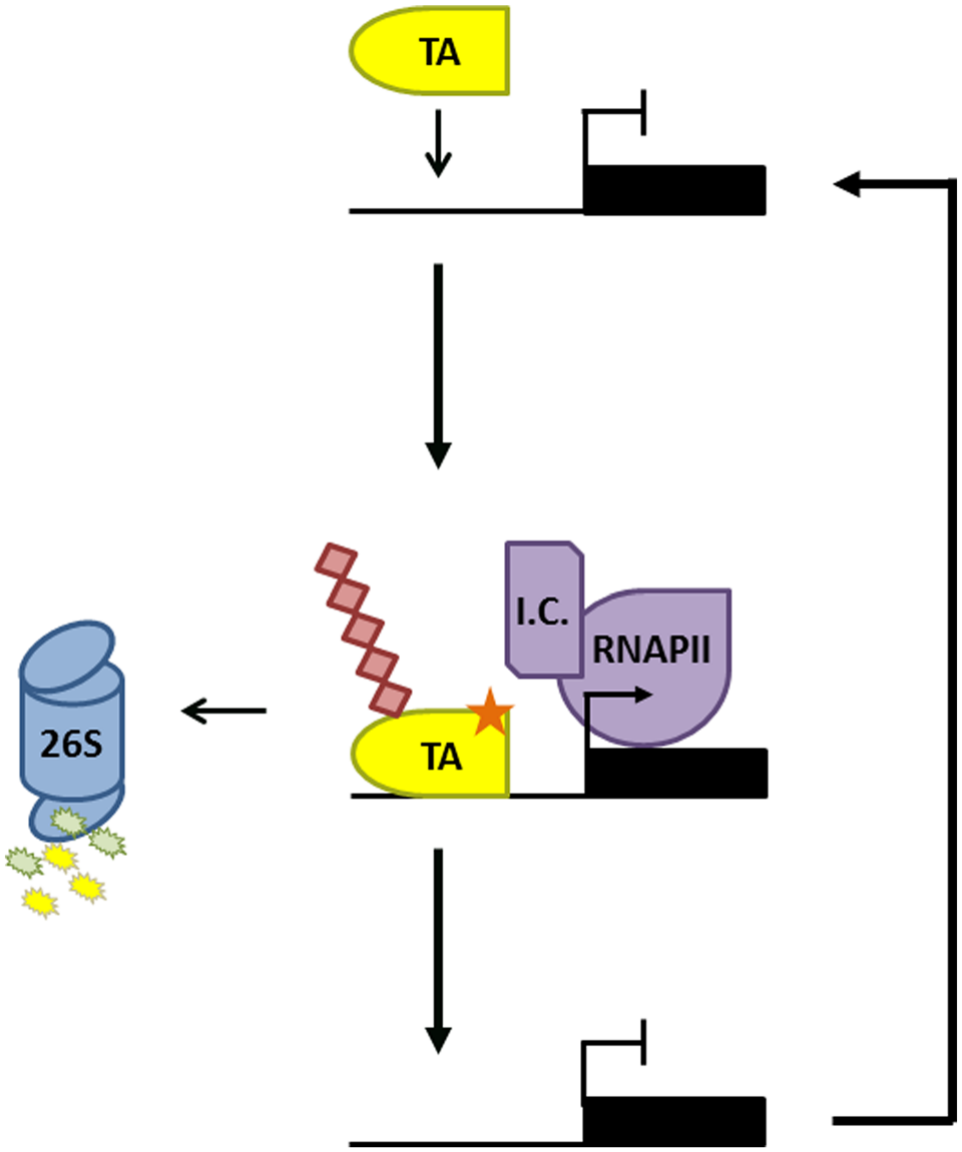
Proteasome-mediated activator turnover activates transcription. Promoter binding of a transcription activator (TA) results in recruitment of the transcription initiation complex (IC) and RNA Polymerase II (RNAPII). The TA is subsequently phosphorylated (orange star) by a kinase within the IC, marking it for ubiquitination (red diamonds) and degradation by the 26S proteasome. This allows a new TA to bind the promoter and reinitiate a new round of gene transcription.

Besides suppression of SA-responsive immune genes, the proteasome is also involved in gene activation. Pharmacological inhibition of the proteasome, genetic mutation of Cullin 3, and mutation of an NPR1 phosphorylation motif all stabilized the NPR1 protein but greatly reduced the SA-induced expression of its target genes in *Arabidopsis* (Spoel et al., 2009). Similarly, SA-induced transcriptional activity of OsWRKY45 in rice was impaired in the presence of proteasome inhibitor (Matsushita et al., 2013). Turnover of OsWRKY45 was dependent on a small 26 amino acid C-terminal region, which importantly was also required for its transactivation activity. Such overlap between transactivation domains and degradation motifs that signal ubiquitin-mediated proteasomal degradation has previously been discovered in transcription activators in both yeast and mammals (Salghetti et al., 2000). Fusion of well-defined degron motifs from yeast cyclin proteins to a DNA-binding domain even auto-activated gene transcription (Salghetti et al., 2000), suggesting that the intrinsic ability to activate transcription also makes activators a target for the ubiquitin-mediated proteasome. Additional work showed that like NPR1 and OsWRKY45, other activators also required turnover to unleash their full transcriptional potential (Spoel et al., 2010; Geng et al., 2012). This transcription process, sometimes dubbed ‘destruction–activation’, has been studied in more detail for GCN4 (General Control Non-inducible 4), a potent activator of genes involved in amino acid homeostasis. Upon amino acid starvation, the CDC4 F-box subunit of SCF^CDC4^ ligase targets GCN4 for ubiquitin-mediated degradation, a process required for recruitment of RNA Polymerase II (RNAPII) to GCN4 target genes (Lipford et al., 2005). Crucially, GCN4 was marked for degradation by the phosphorylative action of SRB10, a cyclin-dependent-kinase associated with the C-terminal domain of RNAPII (Liao et al., 1995; Chi et al., 2001). This indicates that when GCN4 initiates a round of transcription by recruiting RNAPII, it simultaneously triggers its own destruction. These results have led to the hypothesis that transcriptionally ‘spent’ activators may need to be cleared by the proteasome to reset target promoters and allow binding of ‘fresh’ activators (**Figure 1**; Lipford et al., 2005; Kodadek et al., 2006; Geng et al., 2012). A similar mode of regulation may control transcriptional activity of NPR1 and OsWRKY45 in plant immunity, as site-specific phosphorylation of a degron motif in NPR1was necessary for its ubiquitination and degradation, as well as for timely and sustained target gene expression (Spoel et al., 2009, 2010). Intriguingly, transcription initiation by MYC2, a transcription activator responsive to the developmental and immune hormone jasmonic acid, is also regulated by phosphorylation-induced proteasomal degradation (Zhai et al., 2013). These findings imply that proteasome-mediated regulation of transcription activators may be a general mechanism to control gene expression programs in plant immunity.

Elegant studies on the estrogen receptor ERα in mammalian cells have shed more light on why activators are turned over in the process of activating gene transcription. Upon ligand binding, nuclear localized ERα forms a stable dimer, and associates with cofactors on estrogen-responsive DNA elements to trigger gene transcription. Not only did inhibition of ERα proteolysis suppress its transcriptional activity, *vice versa* inhibition of RNAPII prevented degradation of ERα, indicating that activator turnover and transcriptional activity were interdependent (Reid et al., 2003). By following ERα transactivation over fine time scales by chromatin immunoprecipitation, it was proposed that ERα-mediated transcription may have distinct cyclical phases in which the ubiquitin-mediated proteasome plays key roles (Metivier et al., 2003). In this model, the first cycle is transcriptionally non-productive but results in ERα-induced remodeling of the promoter to commit it to transcription. In subsequent cycles ERα orchestrates the ordered recruitment of cofactors, ultimately resulting in gene transcription via recruitment of RNAPII. Importantly, experimental data showed that the proteasome was recruited to an ERα target promoter toward the end of each cycle and preceded the clearance of ERα and general transcription cofactors. Thus, proteasome activity is thought to be vital to allow ERα-dependent promoters to move from the transcriptionally non-productive to productive phase and to permit productive cycles to continue until transcription is no longer required (Metivier et al., 2003; Zhou and Slingerland, 2014). If these findings indeed represent a general model for transcription regulation, then the proteasome could have additional roles in SA-responsive gene transcription in plants, including promoter remodeling and ordered cofactor degradation.

But why would cyclical activation of transcription by unstable activators be advantageous over continuous activation by stable activators? Although the answer to this question remains at large, a recent mathematical and *in silico* analysis of proteasome involvement in transcription may have provided some clues (Lee et al., 2014). The gene targets of many mammalian transcription activators often include components of E3 ligases that promote proteolysis of that activator, generating a negative feedback loop to maintain appropriate levels of activator. Mathematical modeling of this feedback loop showed that cellular perturbations resulting in destabilization of the E3 ligase led to over-accumulation of activators and subsequent hyper-activation of gene expression. However, if the E3 ligase was modeled as a necessary transcription cofactor working in conjunction with the activator, a much more measured gene expression output was achieved upon cellular perturbation. These models suggest that the paradoxical involvement of E3 ligases in gene transcription activated by unstable activators may be necessary to provide a cellular safety mechanism. The authors of this work compared this to the principle of safety interlock devices in engineering, where a system will not function unless safety can be guaranteed (Lee et al., 2014). A similar system may be operational for NPR1- and OsWRKY45-dependent gene expression. Notably, interrogation of a list of NPR1-dependent genes provided by Wang et al. (2006) indicates that NPR1 activates the expression of genes encoding for its paralogues, NPR3, and NPR4. These BTB-containing proteins function as substrate adaptors that recruit NPR1 to CRL3 for ubiquitination and subsequent degradation (Fu et al., 2012). This suggests that similar to the mathematical system described above, a negative feedback loop may exist between NPR1 and CRL3^NPR3/NPR4^. As CRL3 has a supportive role in NPR1-dependent gene transcription (Spoel et al., 2009), it may be part of a cellular safety mechanism to keep NPR1 activity in check when cellular perturbations are encountered. In support of this hypothesis, although genetic perturbations of CRL3^NPR3/NPR4^ activity resulted in autoimmune phenotypes due to over-accumulation of NPR1 protein, this did not lead to over-activation of NPR1 target genes in the presence of SA (Spoel et al., 2009; Fu et al., 2012).

## Processive Ubiquitination of Transcription Activators

**FIGURE 2:**
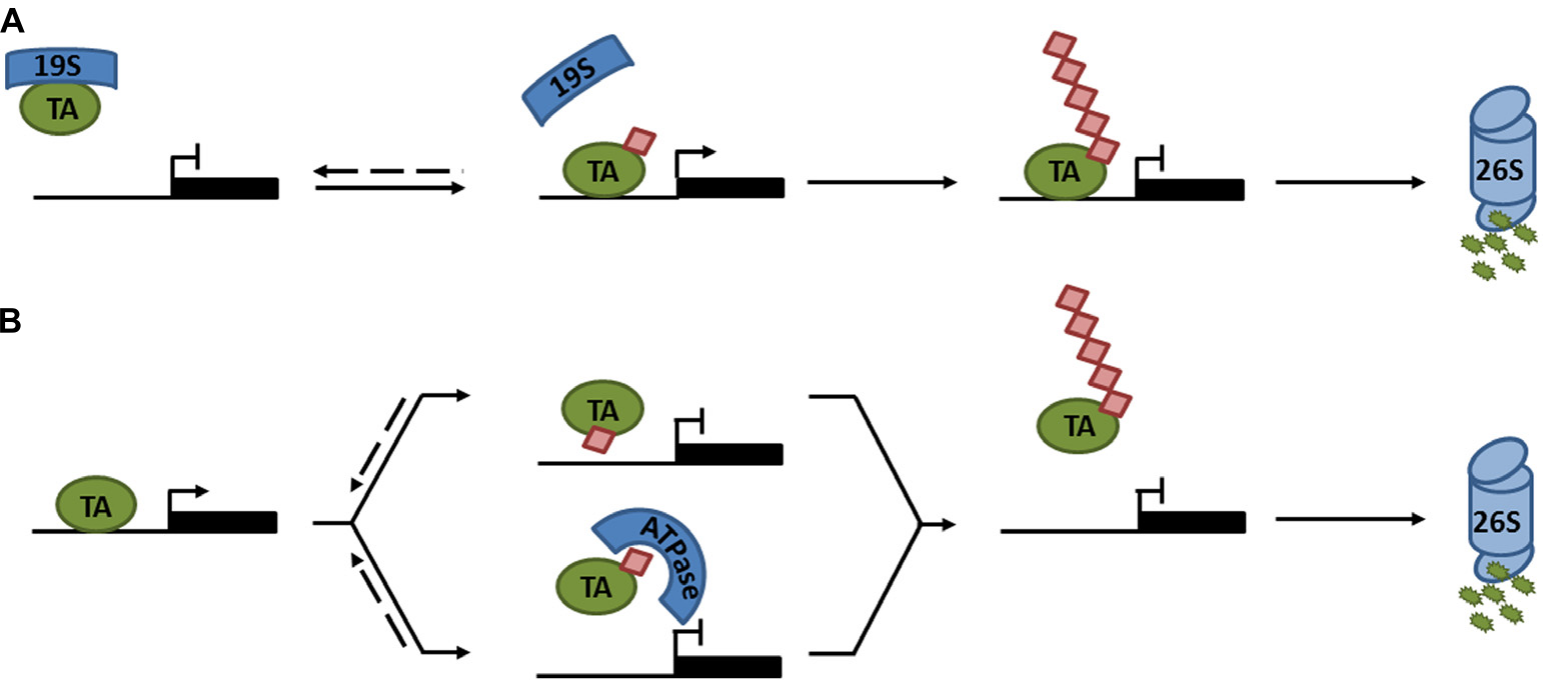
Regulation of transcription activator activity by monoubiquitination. **(A)** The 19S proteasome subcomplex binds an unmodified transcription activator (TA), preventing it from associating with its target promoter. Monoubiquitination of the TA disrupts association with the 19S subcomplex, allowing the TA to bind its target promoter and activate gene expression. Subsequent polyubiquitination marks the TA for degradation by the 26S proteasome. **(B)** Monoubiquitination of a TA prevents transcription either by sterically hindering the binding to its target promoter (top) or by recruiting an ATPase that prevents it from associating with its target promoter (bottom). Subsequent polyubiquitination marks the TA for degradation by the 26S proteasome. Dashed lines indicate reversible steps.

In plants, research has mainly focused on polyubiquitination as a means of regulating protein degradation. However, recent advances in understanding processive ubiquitination in several eukaryotes have highlighted that ubiquitin may have additional important roles in the control of plant transcription factors. The notion that ubiquitin may be directly involved in transcription activation was first explored in yeast. Transcription induced by an artificial activator consisting of the yeast VP16 transactivation domain and the bacterial LexA DNA binding protein (LexA-VP16), was shown to require ubiquitination and degradation mediated by the F-box protein MET30. Strikingly, when ubiquitin was fused in-frame to LexA-VP16, the requirement for MET30 was completely bypassed (Salghetti et al., 2001), suggesting that ubiquitination has dual functions to both activate and destroy transcription activators. Subsequently, additional studies indicated roles for monoubiquitination in transcription activation (Bres et al., 2003; Greer et al., 2003; Burgdorf et al., 2004). Monoubiquitination does not usually signal for proteasome-mediated degradation, for which approximately four or more Lys48-linked ubiquitins are required (Thrower et al., 2000). Instead it was reported that promoter occupancy of the yeast prototypical transcription activator, GAL4, was stabilized by monoubiquitination (Ferdous et al., 2007; Archer et al., 2008b). Interestingly, unmodified GAL4 was destabilized by ATPase activity of the proteasome 19S regulatory particle, preventing transcription activation. Monoubiquitination limited the lifetime of physical interactions between the GAL4 activation domain and 19S subunits (**Figure 2A**; Archer et al., 2008a). This type of regulatory system likely extends to many other eukaryotes, as interactions between tumor suppressor protein p53, a transcription activator in mammalian cells, and its target promoters were also destabilized by 19S ATPases (Kim et al., 2009).

In contrast to these reports, examples of monoubiquitination leading to suppression of transcription activators have also emerged (Peloponese et al., 2004; Inui et al., 2011; Tang et al., 2011; Ndoja et al., 2014). Using the artificial LexA-VP16 activator described above, a recent report argued that in-frame fusion of ubiquitin to LexA-VP16 was susceptible to cleavage by deubiquitinases (DUBs). Preventing deubiquitination by introducing a non-cleavable ubiquitin-LexA-VP16 mutant resulted in suppression of transcriptional activity by the AAA^+^ ATPase, CDC48, which stripped this activator from its target promoter (**Figure 2B**; Ndoja et al., 2014). These findings were extended from artificial to native transcription activators. CDC48 was implicated in yeast sulfur metabolism by removing the monoubiquitinated transcriptional activator, MET4, from its target promoters upon ubiquitination by SCF^MET30^ (Ndoja et al., 2014). Moreover, monoubiquitination of mammalian receptor-activated SMADs (R-SMAD), involved in TGF-β-mediated embryonic development and tissue homeostasis, attenuated its transcriptional activity by two possible mechanisms: (i) monoubiquitination prevented either R-SMAD transcription complex formation or DNA binding by steric hindrance; or (ii) the CDC48 homolog, p97, actively removed monoubiquitinated R-SMADs from the promoter (**Figure 2B**; Inui et al., 2011; Tang et al., 2011; Ndoja et al., 2014). Taken together, all these reports clearly illustrate that monoubiquitination can directly regulate the activity of transcription activators through a variety of different mechanisms (**Figure 2**). Additionally, monoubiquitination may indirectly regulate the activities of some activators by modulating their nucleocytoplasmic localization (van der Horst et al., 2006).

While monoubiquitination may play a regulatory role, processive ubiquitin chain elongation subsequently leads to activator turnover (Kodadek et al., 2006). This processive mono-to-polyubiquitination switch was explored in particular detail for the human Steroid Receptor Coactivator-3 (SRC-3). SRC-3 is an important developmental transcription coactivator, whose uncontrolled expression can lead to oncogenesis. SRC-3 was found to be subject to phosphorylation-dependent polyubiquitination by SCF^Fbw7α^, resulting in its transcription-coupled degradation. However, SRC-3 was also multi (mono)-ubiquitinated by SCF^Fbw7α^, which enhanced its transcriptional activity. Hence, it was proposed that biphasic, processive ubiquitination (i.e., transitioning from mono- to polyubiquitination) generates a timer for the functional lifetime of SRC-3 (Wu et al., 2007).

These intriguing findings relating to eukaryotic transcription indicate that ubiquitin-mediated control of transcription (co)activators in SA-dependent immunity is far more complex than generally appreciated. Current efforts in this field by several labs, including our own, may soon reveal additional roles for ubiquitin and ubiquitin ligases in the transcription activation of immune genes.

## CRL3-Mediated Degradation of SA-Responsive Repressors?

In the past decade intimate relationships between plant hormone signaling and the ubiquitin-mediated proteasome have been uncovered. Recurring roles for CRL1 and CRL3 are found in jasmonic acid, ethylene, auxin, gibberellin, abscisic acid, strigolactone, and zeatin signaling (Kelley and Estelle, 2012). The role of CRL1 in jasmonic acid- and auxin-responsive gene expression is especially similar. In both cases the hormone facilitates physical interaction of the CRL1 F-box subunit with transcriptional repressors to form a hormone coreceptor complex. Hormone-dependent recruitment of repressors to CRL1 leads to their poly-ubiquitination and degradation, releasing the activity of transcriptional activators (Kelley and Estelle, 2012). A strikingly similar hormone perception mechanism regulates SA signaling, but instead utilizes CRL3. The CRL3 substrate adaptors NPR3 and NPR4 were shown to act as SA receptors. Whereas SA binding facilitated interaction between NPR3 and NPR1, it disrupted NPR4-NPR1 interaction. Moreover, genetic deletion of *NPR3* and *NPR4* severely impaired the ability to coimmunoprecipitate Cullin 3 and NPR1, indicating that NPR1 is the substrate of an SA-sensitive CRL3^NPR3/NPR4^ (Fu et al., 2012).

It is likely that CRL3 complexes exist with roles that extend beyond targeting NPR1. In analogy to jasmonic acid and auxin signaling, CRL3 could target a number of transcription (co)repressors described for SA-responsive genes. For example, TGA2 transcription factors act as repressors of *PR* genes (Zhang et al., 2003; Kesarwani et al., 2007). Moreover, NPR3 and NPR4 physically interact with TGA2 and other members of the TGA family (Liu et al., 2005; Zhang et al., 2006; Shi et al., 2013), implying that a CRL3^NPR3/4^ might target TGA factors for degradation to activate SA-responsive genes. Other conceivable targets of CRL3 include SNI1 (Suppressor of NPR1, Inducible), a corepressor of mostly NPR1-depenent genes (Mosher et al., 2006). SNI1 was recently shown to associate with CBNAC, a calmodulin-binding NAC transcription factor. Genetic analysis suggested that CBNAC is a transcription repressor of SA-dependent immune responses. Interestingly, SNI1 facilitated the binding of CBNAC to a DNA-binding motif in the SA-responsive *PR-1* promoter (Kim et al., 2012). Finally, several NPR1-interacting NIMIN (NIM1/NPR1-Interacting) proteins act as corepressors, and their removal or inactivation is presumable necessary for activation of SA-responsive gene expression (Weigel et al., 2005). Thus, CRL3 targets could include SNI1, CBNAC, TGA factors, and NIMINs, but little is currently known about the stability of these (co)repressors. Analysis of transcription (co)factor interaction networks in rice between OsNPR paralogues, TGA factors, and NRR (Negative Regulator of Resistance) proteins that share limited homology to *Arabidopsis* NIMINs, paint a similar picture (Chern et al., 2014). All these factors formed a wide network of interactions in both yeast two-hydrid and split YFP assays, suggesting that involvement of CRL3 complexes in immunity may be functionally conserved in rice.

Alternative to direct targeting of (co)repressors by CRL3, a recent report suggests that these ubiquitin ligases can also promote the concurrent ubiquitination of multiple associated substrates. Upon light induction, the transcription factor PIF3 is recruited to CRL3^LRB^ for ubiquitination. Strikingly, it was found that the PIF3 interaction partner, PhyB, was concomitantly recruited by CRL3^LRB^ (Ni et al., 2014). CRL3 dimerisation through BTB domains might facilitate concurrent substrate degradation, essentially bringing together two active sites for substrate ubiquitination (Stogios et al., 2007). Hence, it plausible that CRL3^NPR3/NPR4^ simultaneously targets complexes consisting of NPR1 and the transcriptional repressors that physically interact with NPR1.

Peculiarly, unlike NPR3 and NPR4, NPR1 has not yet been observed in the substrate adaptor position of a CRL3 (Dieterle et al., 2005; Fu et al., 2012). However, computational predictions of NPR1 protein structure suggest that it forms a typical BTB domain fold that should allow interaction with Cullin 3 (Tada et al., 2008). Moreover, immediately C-terminal to the BTB domain, NPR1, NPR3, and NPR4 all contain key elements of a conserved helical 3-box structure that, analogous to the F-box motif, was shown to stimulate Cullin 3 interaction by packing tightly against its N-terminus (Zhuang et al., 2009; Canning et al., 2013). Reports that NPR1 itself may directly sense SA or may also be a SA receptor (Maier et al., 2011; Wu et al., 2012) further suggests that NPR1 could be part of a CRL analogous to other plant hormone pathways, although definitive proof for NPR1 as an SA receptor was not supported by another study (Fu et al., 2012).

**FIGURE 3:**
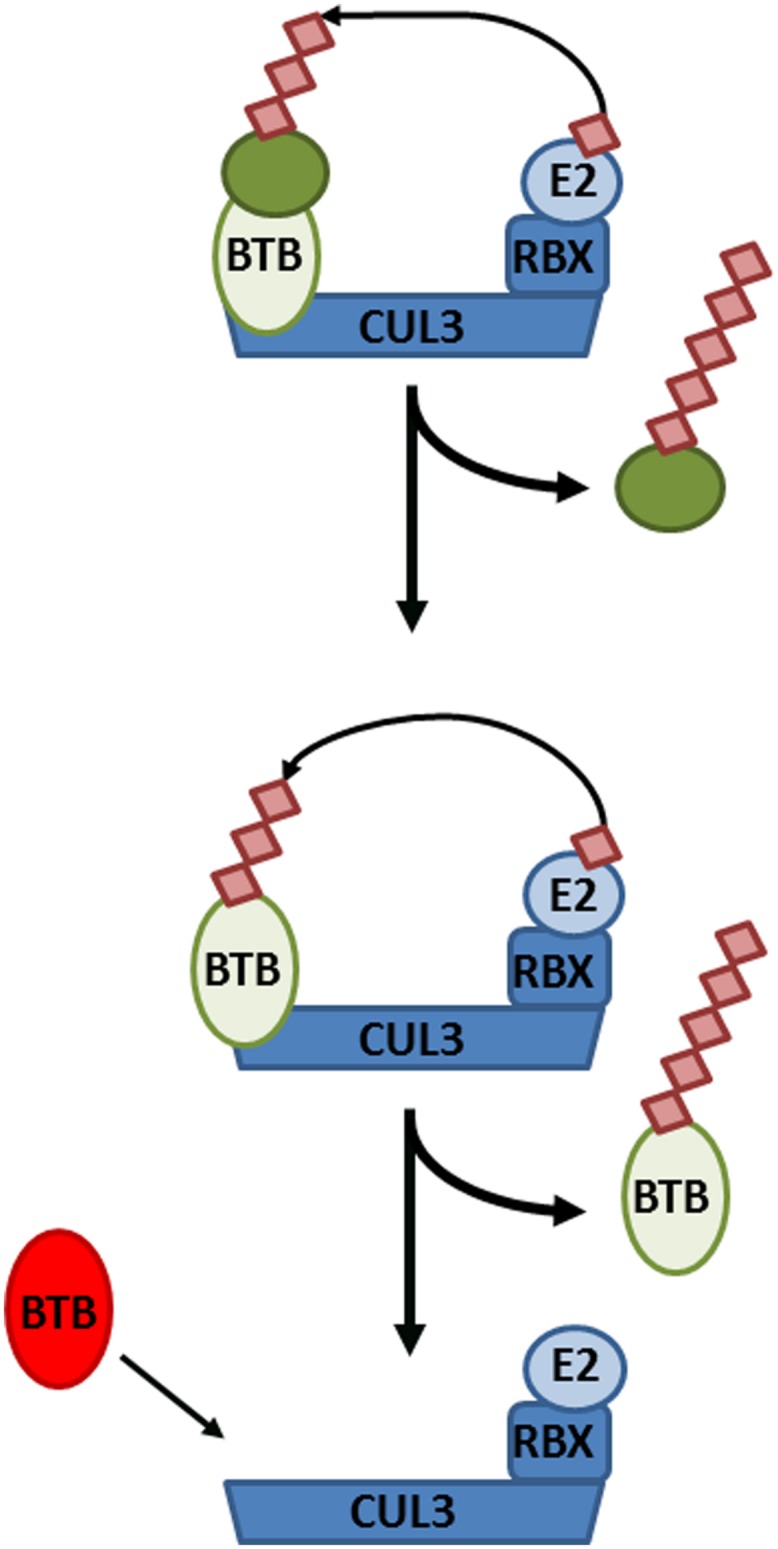
Cullin-RING ligase 3 (CRL3) autoubiquitination and adaptor switching. **(Top)** A target substrate (dark green circle) is polyubiquitinated and targeted for degradation by CRL3, consisting of the CUL3 backbone, Bric-à-brac, Tramtrack, and Broad complex (BTB) domain-containing adaptor, RING-Box protein (RBX), and an E2 conjugating enzyme. **(Middle)** After all available substrates have been polyubiquitinated and degraded, the BTB adaptor itself becomes subject to autoubiquitination and degradation. **(Bottom)** Consequently, the CRL3 can now switch to a new BTB adaptor in order to polyubiquitinate different substrates.

If NPR1 does indeed reside in a substrate adaptor position of CRL3, this would have important implications for the role of its own turnover in SA-responsive gene expression. First, this would create an additional layer of complexity whereby a CRL3^NPR3/NPR4^ regulates the formation of a CRL3^NPR1^. Secondly, CRL substrate adaptors often paradoxically exhibit instability themselves. In absence of a substrate, both F-box and BTB adaptors have been shown to be subject to auto-ubiquitination within their respective CRLs (Bosu and Kipreos, 2008). The necessity of NPR1 turnover in activation of SA-responsive genes may therefore reflect a requirement to allow switching of diverse NPR substrate adaptors within core CRL3 complexes (**Figure 3**).

## CRLs in SA-Mediated Programmed Cell Death and Survival

**FIGURE 4:**
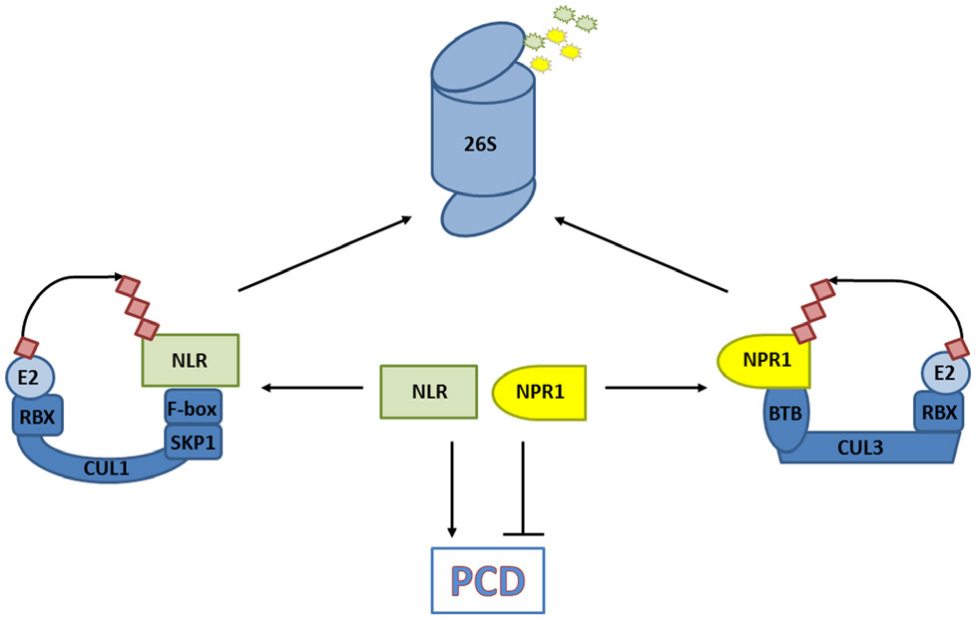
CRLs decide on cell fate. The degradation of substrates that promote programmed cell death (PCD) in response to a pathogen [e.g., nucleotide-binding/leucine-rich repeat domain containing (NLR) immune receptors], and those that prevent PCD (e.g., non-expresser of PR genes, NPR1) are controlled by CRLs. The balance of substrate abundance between promoters and suppressors of PCD dictates cell fate, and is regulated by their ubiquitination and 26S proteasome-mediated degradation.

Salicylic acid is an agonist of PCD responses induced by NLR immune receptors upon intracellular detection of pathogen effectors. In some cases cellular decisions to live or die upon pathogen infection are shaped by the activities of CRLs. Mutation of CRL3^NPR3/NPR4^ components suggested that the stability of its substrate, NPR1, is an important determinant in PCD induced by the NLR receptors RPS2 and RPM1 (Fu et al., 2012). Indeed, analysis of pathogen-induced PCD in *npr1* mutants previously revealed that NPR1 suppressed PCD induced by these NLR receptors (Rate and Greenberg, 2001). Moreover, mutation of *NPR1* partially restored RPS2- and RPM1-induced PCD in *npr3* and *npr4* mutants (Fu et al., 2012). These results indicate that elevated levels of NPR1 promote cell survival and that its removal by CRL3^NPR3/NPR4^ is required for successful PCD induced by at least some NLR receptor classes (**Figure 4**). In agreement with its role in promoting cell survival, genetic experiments have indicated that the presence of NPR1 is not essential for successful NLR receptor-induced PCD and immunity (Rairdan and Delaney, 2002).

In contrast to NPR1’s pro-survival role, NLR receptors instigate PCD responses upon perception of pathogen effectors. In the absence of pathogen threats, NLR receptors must be kept tightly controlled to avoid autoimmune responses. Overexpression of the tomato NLR receptor, Prf, resulted in strong autoimmune phenotypes, including elevated SA levels and expression of SA-responsive genes in absence of pathogen attack (Oldroyd and Staskawicz, 1998). Moreover, overexpression of NLR receptors due to genomic duplication in the *Arabidopsis bal* variant also led to constitutive SA responses in absence of a pathogen as well as morphological defects such as severely stunted growth, highlighting the trade-off between growth and defense (Stokes et al., 2002; Yi and Richards, 2009). Similarly, mutation of the potential transcription corepressor, SRFR1, resulted in autoimmunity due to transcriptional upregulation of the co-regulated NLR receptors SNC1, RPS2, and RPS4 (Kwon et al., 2009; Kim et al., 2010; Li et al., 2010). Collectively these examples illustrate that the cellular protein levels of some NLR receptors are linked to their immune activities.

Recent work revealed that protein levels of several NLR receptors are tightly controlled by CRL activities. An early screen for mutations leading to SA-mediated autoimmune phenotypes identified the *cpr1* (*constitutive expressor of PR genes*) mutant (Bowling et al., 1994). Importantly, protein levels of the NLR receptors SNC1 and RPS2 were inversely correlated with CPR1 activity, and loss-of-function mutations in *SNC1* largely suppressed the autoimmune phenotype of mutant *cpr1* plants. Cloning of *CPR1* revealed it encodes an F-box protein, suggesting it controls the abundance of specific NLR receptors by targeting them for proteasome-mediated degradation. Indeed, CPR1 directly interacted with SNC1 and RPS2, and in case of SNC1 this appeared to lead to its polyubiquitination and degradation by the proteasome (Cheng et al., 2011; Gou et al., 2012). NLR receptor signaling probably involves other CRL1 ubiquitin ligases as well but with distinct functions. Rather than eliciting autoimmunity, silencing of the F-box protein ACIF1 in tobacco and tomato compromised NLR receptor-mediated PCD and immunity (van den Burg et al., 2008). ACIF1 interacted with other CRL1 subunits, suggesting it can form a functional ubiquitin ligase but its direct targets remain unknown. Notably, several non-CRL ubiquitin ligases that regulate NLR accumulation or signaling have also been identified and are discussed in other excellent reviews (Marino et al., 2012; Duplan and Rivas, 2014). Hence, ubiquitin ligases – and CRLs in particular – play an integral role in cellular decisions of life and death by controlling the level of NLR receptors and PCD suppressors (**Figure 4**).

In addition to canonical ubiquitination pathway enzymes (E1, E2, E3), an E4 class of ubiquitin ligases has been described (Koegl et al., 1999). E4 ligases also polyubiquitinate substrates, but contrary to E3 ligases, they largely lack substrate specificity and rather function to elongate existing ubiquitin chains, thereby potentially promoting recognition of substrates by the proteasome. A recent forward genetic screen for mutants that enhanced autoimmunity of *snc1*, a mutation that renders this NLR receptor constitutively active, identified the E4 ligase MUSE3 (Mutant *snc1*-Enhancing; Huang et al., 2014). Mutant *muse3* plants exhibited elevated levels of SNC1 and RPS2, while overexpression of MUSE3 in itself did not lead to reduction of NLR receptor accumulation. However, coexpression of MUSE3 together with the F-box protein CPR1 resulted in a greater decrease in NLR receptor accumulation than observed with CPR1 expression alone, indicating that MUSE3 and SCF^CPR1^ function cooperatively to destabilize NLR receptors. In case of SNC1 but not RPS2, a direct physical association with MUSE3 was indeed found, suggesting that MUSE3 may recognize NLR receptors via distinct mechanisms. Thus, an intricate set of cooperative ubiquitin ligases underpin SA-dependent NLR receptor signaling to prevent autoimmunity and promote timely activation of immune responses.

## The Road Ahead…

In this review we have discussed the emerging roles of ubiquitin ligases in aspects of SA-mediated immune signaling, including transcriptional reprogramming and cellular decisions of life and death. Similar to other hormone signaling pathways, members of the CRL class of ubiquitin ligases appear to fulfill particularly important tasks, although the targets of these CRLs still remain largely unknown. In SA-induced gene transcription the precise role of CRL3-mediated turnover of NPR1 requires further investigation into processive ubiquitination events and it remains to be discovered if immune-induced CRL3 targets substrates other than NPR1 for proteasome-mediated degradation. Although the role of CRLs in controlling the accumulation of specific NLR receptors is becoming increasingly clear, it remains poorly understood why protein abundance is a key factor in determining NLR receptor activity. Conformational control of NLR receptors by highly conserved eukaryotic chaperone complexes is thought to keep receptors in a recognition-competent state and facilitate their activation upon pathogen perception (Shirasu, 2009; van Ooijen et al., 2010). It is plausible that uncontrolled accumulation of some NLR receptors could result in a shortage of available chaperones and consequent conformation-induced auto-activation of NLR receptors. Finally, many E3 ligases construct ubiquitin chain topologies distinct from proteasome-recognized Lys48-linkages. These alternative chain topologies serve a wide variety of different cellular signaling functions in eukaryotes, yet little is known about their existence and roles in plant biology (Walsh and Sadanandom, 2014). Hence, much remains to be discovered in the exciting field of plant ubiquitin signaling in general and in SA-mediated immune responses in particular.

## References

[B1] Aravind L. Koonin E. V. (1999). Fold prediction and evolutionary analysis of the POZ domain: structural and evolutionary relationship with the potassium channel tetramerization domain. *J. Mol. Biol.* 285 1353–1361 10.1006/jmbi.1998.2394 9917379

[B2] Archer C. T. Burdine L. Liu B. Ferdous A. Johnston S. A. Kodadek T. (2008a). Physical and functional interactions of monoubiquitylated transactivators with the proteasome. *J. Biol. Chem.* 283 21789–21798 10.1074/jbc.M803075200 18515799 PMC2490782

[B3] Archer C. T. Delahodde A. Gonzalez F. Johnston S. A. Kodadek T. (2008b). Activation domain-dependent monoubiquitylation of Gal4 protein is essential for promoter binding in vivo. *J. Biol. Chem.* 283 12614–12623 10.1074/jbc.M801050200 18326036 PMC2335349

[B4] Bosu D. R. Kipreos E. T. (2008). Cullin-RING ubiquitin ligases: global regulation and activation cycles. *Cell Div.* 3 7 10.1186/1747-1028-3-7 PMC2266742 18282298

[B5] Bowling S. A. Guo A. Cao H. Gordon A. S. Klessig D. F. Dong X. (1994). A mutation in *Arabidopsis* that leads to constitutive expression of systemic acquired resistance. *Plant Cell* 6 1845–1857 10.1105/tpc.6.12.1845 7866028 PMC160566

[B6] Boyle P. Le Su E. Rochon A. Shearer H. L. Murmu J. Chu J. Y. (2009). The BTB/POZ domain of the *Arabidopsis* disease resistance protein NPR1 interacts with the repression domain of TGA2 to negate its function. *Plant Cell* 21 3700–3713 10.1105/tpc.109.069971 19915088 PMC2798319

[B7] Bres V. Kiernan R. E. Linares L. K. Chable-Bessia C. Plechakova O. Treand C. (2003). A non-proteolytic role for ubiquitin in Tat-mediated transactivation of the HIV-1 promoter. *Nat. Cell Biol.* 5 754–761 10.1038/ncb1023 12883554

[B8] Burgdorf S. Leister P. Scheidtmann K. H. (2004). TSG101 interacts with apoptosis-antagonizing transcription factor and enhances androgen receptor-mediated transcription by promoting its monoubiquitination. *J. Biol. Chem.* 279 17524–17534 10.1074/jbc.M313703200 14761944

[B9] Canning P. Cooper C. D. O. Krojer T. Murray J. W. Pike A. C. Chaikuad A. (2013). Structural basis for Cul3 protein assembly with the BTB-Kelch family of E3 ubiquitin ligases. *J. Biol. Chem.* 288 7803–7814 10.1074/jbc.M112.437996 23349464 PMC3597819

[B10] Cao H. Bowling S. A. Gordon A. S. Dong X. (1994). Characterization of an *Arabidopsis* mutant that is nonresponsive to inducers of systemic acquired resistance. *Plant Cell* 6 1583–1592 10.1105/tpc.6.11.1583 12244227 PMC160545

[B11] Cao H. Glazebrook J. Clarke J. D. Volko S. Dong X. (1997). The *Arabidopsis* NPR1 gene that controls systemic acquired resistance encodes a novel protein containing ankyrin repeats. *Cell* 88 57–63 10.1016/S0092-8674(00)81858-9 9019406

[B12] Cheng Y. T. Li Y. Huang S. Huang Y. Dong X. Zhang Y. (2011). Stability of plant immune-receptor resistance proteins is controlled by SKP1-CULLIN1-F-box (SCF)-mediated protein degradation. *Proc. Natl. Acad. Sci. U.S.A.* 108 14694–14699 10.1073/pnas.1105685108 21873230 PMC3167521

[B13] Chern M. Bai W. Ruan D. Oh T. Chen X. Ronald P. C. (2014). Interaction specificity and coexpression of rice NPR1 homologs 1 and 3 (NH1 and NH3), TGA transcription factors and Negative Regulator of Resistance (NRR) proteins. *BMC Genomics* 15:461 10.1186/1471-2164-15-461 PMC4094623 24919709

[B14] Chi Y. Huddleston M. J. Zhang X. Young R. A. Annan R. S. Carr S. A. (2001). Negative regulation of Gcn4 and Msn2 transcription factors by Srb10 cyclin-dependent kinase. *Genes Dev.* 15 1078–1092 10.1101/gad.867501 11331604 PMC312682

[B15] Delaney T. P. Friedrich L. Ryals J. A. (1995). Arabidopsis signal transduction mutant defective in chemically and biologically induced disease resistance. *Proc. Natl. Acad. Sci. U.S.A.* 92 6602–6606 10.1073/pnas.92.14.6602 11607555 PMC41566

[B16] Delaney T. P. Uknes S. Vernooij B. Friedrich L. Weymann K. Negrotto D. (1994). A central role of salicylic acid in plant disease resistance. *Science* 266 1247–1250 10.1126/science.266.5188.1247 17810266

[B17] Després C. DeLong C. Glaze S. Liu E. Fobert P. R. (2000). The *Arabidopsis* NPR1/NIM1 protein enhances the DNA binding activity of a subgroup of the TGA family of bZIP transcription factors. *Plant Cell* 12 279–290 10.1105/tpc.12.2.279 10662863 PMC139764

[B18] Dieterle M. Thomann A. Renou J. P. Parmentier Y. Cognat V. Lemonnier G. (2005). Molecular and functional characterization of *Arabidopsis* Cullin 3A. *Plant J.* 41 386–399 10.1111/j.1365-313X.2004.02302.x 15659098

[B19] Duplan V. Rivas S. (2014). E3 ubiquitin-ligases and their target proteins during the regulation of plant innate immunity. *Front. Plant Sci.* 5:42 10.3389/fpls.2014.00042 PMC3923142 24592270

[B20] Ferdous A. Sikder D. Gillette T. Nalley K. Kodadek T. Johnston S. A. (2007). The role of the proteasomal ATPases and activator monoubiquitylation in regulating Gal4 binding to promoters. *Genes Dev.* 21 112–123 10.1101/gad.1493207 17167105 PMC1759896

[B21] Fu Z. Q. Dong X. (2013). Systemic acquired resistance: turning local infection into global defense. *Annu. Rev. Plant Biol.* 64 839–863 10.1146/annurev-arplant-042811-105606 23373699

[B22] Fu Z. Q. Yan S. Saleh A. Wang W. Ruble J. Oka N. (2012). NPR3 and NPR4 are receptors for the immune signal salicylic acid in plants. *Nature* 486 228–232 10.1038/nature11162 22699612 PMC3376392

[B23] Geng F. Wenzel S. Tansey W. P. (2012). Ubiquitin and proteasomes in transcription. *Annu. Rev. Biochem.* 81 177–201 10.1146/annurev-biochem-052110-120012 22404630 PMC3637986

[B24] Glazebrook J. Rogers E. E. Ausubel F. M. (1996). Isolation of *Arabidopsis* mutants with enhanced disease susceptibility by direct screening. *Genetics* 143 973–982. 8725243 10.1093/genetics/143.2.973 PMC1207353

[B25] Gou M. Shi Z. Zhu Y. Bao Z. Wang G. Hua J. (2012). The F-box protein CPR1/CPR30 negatively regulates R protein SNC1 accumulation. *Plant J.* 69 411–420 10.1111/j.1365-313X.2011.04799.x 21967323

[B26] Greer S. F. Zika E. Conti B. Zhu X. S. Ting J. P. (2003). Enhancement of CIITA transcriptional function by ubiquitin. *Nat. Immunol.* 4 1074–1082 10.1038/ni985 14528304

[B27] Huang Y. Minaker S. Roth C. Huang S. Hieter P. Lipka V. (2014). An E4 ligase facilitates polyubiquitination of plant immune receptor resistance proteins in *Arabidopsis*. *Plant Cell* 26 485–496 10.1105/tpc.113.119057 24449689 PMC3963591

[B28] Inui M. Manfrin A. Mamidi A. Martello G. Morsut L. Soligo S. (2011). USP15 is a deubiquitylating enzyme for receptor-activated SMADs. *Nat. Cell Biol.* 13 1368–1375 10.1038/ncb2346 21947082

[B29] Jones J. D. G. Dangl J. L. (2006). The plant immune system. *Nature* 444 323–329 10.1038/nature05286 17108957

[B30] Kelley D. R. Estelle M. (2012). Ubiquitin-mediated control of plant hormone signaling. *Plant Physiol.* 160 47–55 10.1104/pp.112.200527 22723083 PMC3440220

[B31] Kesarwani M. Yoo J. Dong X. (2007). Genetic interactions of TGA transcription factors in the regulation of pathogenesis-related genes and disease resistance in *Arabidopsis*. *Plant Physiol.* 144 336–346 10.1104/pp.106.095299 17369431 PMC1913812

[B32] Kim H. S. Park H. C. Kim K. E. Jung M. S. Han H. J. Kim S. H. (2012). A NAC transcription factor and SNI1 cooperatively suppress basal pathogen resistance in *Arabidopsis thaliana*. *Nucleic Acids Res.* 40 9182–9192 10.1093/nar/gks683 22826500 PMC3467076

[B33] Kim S. H. Gao F. Bhattacharjee S. Adiasor J. A. Nam J. C. Gassmann W. (2010). The *Arabidopsis* resistance-like gene SNC1 is activated by mutations in SRFR1 and contributes to resistance to the bacterial effector AvrRps4. *PLoS Pathog.* 6:e1001172 10.1371/journal.ppat.1001172 PMC2973837 21079790

[B34] Kim Y. C. Wu S. Y. Lim H. S. Chiang C. M. Kodadek T. (2009). Non-proteolytic regulation of p53-mediated transcription through destabilization of the activator∙promoter complex by the proteasomal ATPases. *J. Biol. Chem.* 284 34522–34530 10.1074/jbc.M109.017277 19846554 PMC2787313

[B35] Kinkema M. Fan W. Dong X. (2000). Nuclear localization of NPR1 is required for activation of PR gene expression. *Plant Cell* 12 2339–2350 10.1105/tpc.12.12.2339 11148282 PMC102222

[B36] Kodadek T. Sikder D. Nalley K. (2006). Keeping transcriptional activators under control. *Cell* 127 261–264 10.1016/j.cell.2006.10.002 17055428

[B37] Koegl M. Hoppe T. Schlenker S. Ulrich H. D. Mayer T. U. Jentsch S. (1999). A novel ubiquitination factor, E4 is involved in multiubiquitin chain assembly. *Cell* 96 635–644 10.1016/S0092-8674(00)80574-7 10089879

[B38] Komander D. Rape M. (2012). The ubiquitin code. *Annu. Rev. Biochem.* 81 203–229 10.1146/annurev-biochem-060310-170328 22524316

[B39] Kwon S. I. Kim S. H. Bhattacharjee S. Noh J. J. Gassmann W. (2009). SRFR1 a suppressor of effector-triggered immunity, encodes a conserved tetratricopeptide repeat protein with similarity to transcriptional repressors. *Plant J.* 57 109–119 10.1111/j.1365-313X.2008.03669.x 18774967

[B40] Lee D. Kim M. Cho K. H. (2014). A design principle underlying the paradoxical roles of E3 ubiquitin ligases. *Sci. Rep.* 4 5573 10.1038/srep05573 PMC5381699 24994517

[B41] Li Y. Li S. Bi D. Cheng Y. T. Li X. Zhang Y. (2010). SRFR1 negatively regulates plant NB-LRR resistance protein accumulation to prevent autoimmunity. *PLoS Pathog.* 6:e1001111 10.1371/journal.ppat.1001111 PMC2940742 20862316

[B42] Liao S. M. Zhang J. Jeffery D. A. Koleske A. J. Thompson C. M. Chao D. M. (1995). A kinase-cyclin pair in the RNA polymerase II holoenzyme. *Nature* 374 193–196 10.1038/374193a0 7877695

[B43] Lipford J. R. Smith G. T. Chi Y. Deshaies R. J. (2005). A putative stimulatory role for activator turnover in gene expression. *Nature* 438 113–116 10.1038/nature04098 16267558

[B44] Liu G. Holub E. B. Alonso J. M. Ecker J. R. Fobert P. R. (2005). An *Arabidopsis* NPR1-like gene, NPR4 is required for disease resistance. *Plant J.* 41 304–318 10.1111/j.1365-313X.2004.02296.x 15634206

[B45] Macho A. P. Zipfel C. (2014). Plant PRRs and the activation of innate immune signaling. *Mol. Cell* 54 263–272 10.1016/j.molcel.2014.03.028 24766890

[B46] Maier F. Zwicker S. Hückelhoven A. Meissner M. Funk J. Pfitzner A. J. P. (2011). NONEXPRESSOR OF PATHOGENESIS-RELATED PROTEINS1 (NPR1) and some NPR1-related proteins are sensitive to salicylic acid. *Mol. Plant Pathol.* 12 73–91 10.1111/j.1364-3703.2010.00653.x 21118350 PMC6640455

[B47] Marino D. Peeters N. Rivas S. (2012). Ubiquitination during plant immune signaling. *Plant Physiol.* 160 15–27 10.1104/pp.112.199281 22689893 PMC3440193

[B48] Matsushita A. Inoue H. Goto S. Nakayama A. Sugano S. Hayashi N. (2013). Nuclear ubiquitin proteasome degradation affects WRKY45 function in the rice defense program. *Plant J.* 73 302–313 10.1111/tpj.12035 PMC3558880 23013464

[B49] Metivier R. Penot G. Hubner M. R. Reid G. Brand H. Kos M. (2003). Estrogen receptor-α directs ordered, cyclical, and combinatorial recruitment of cofactors on a natural target promoter. *Cell* 115 751–763 10.1016/S0092-8674(03)00934-6 14675539

[B50] Moore J. W. Loake G. J. Spoel S. H. (2011). Transcription dynamics in plant immunity. *Plant Cell* 23 2809–2820 10.1105/tpc.111.087346 21841124 PMC3180793

[B51] Mosher R. A. Durrant W. E. Wang D. Song J. Dong X. (2006). A comprehensive structure-function analysis of *Arabidopsis* SNI1 defines essential regions and transcriptional repressor activity. *Plant Cell* 18 1750–1765 10.1105/tpc.105.039677 16766691 PMC1488919

[B52] Mou Z. Fan W. Dong X. (2003). Inducers of plant systemic acquired resistance regulate NPR1 function through redox changes. *Cell* 113 935–944 10.1016/S0092-8674(03)00429-X 12837250

[B53] Nakayama A. Fukushima S. Goto S. Matsushita A. Shimono M. Sugano S. (2013). Genome-wide identification of WRKY45-regulated genes that mediate benzothiadiazole-induced defense responses in rice. *BMC Plant Biol.* 13:150 10.1186/1471-2229-13-150 PMC3850545 24093634

[B54] Ndoja A. Cohen R. E. Yao T. (2014). Ubiquitin signals proteolysis-independent stripping of transcription factors. *Mol. Cell* 53 893–903 10.1016/j.molcel.2014.02.002 24613342 PMC4005849

[B55] Ni W. Xu S. L. Tepperman J. M. Stanley D. J. Maltby D. A. Gross J. D. (2014). A mutually assured destruction mechanism attenuates light signaling in *Arabidopsis*. *Science* 344 1160–1164 10.1126/science.1250778 24904166 PMC4414656

[B56] Oldroyd G. E. Staskawicz B. J. (1998). Genetically engineered broad-spectrum disease resistance in tomato. *Proc. Natl. Acad. Sci. U.S.A.* 95 10300–10305 10.1073/pnas.95.17.10300 9707642 PMC21503

[B57] Peloponese J. M. Jr. Iha H. Yedavalli V. R. K. Miyazato A. Li Y. Haller K. (2004). Ubiquitination of human T-cell leukemia virus type 1 tax modulates its activity. *J. Virol.* 78 11686–11695 10.1128/JVI.78.21.11686-11695.2004 15479810 PMC523283

[B58] Petroski M. D. Deshaies R. J. (2005). Function and regulation of Cullin-RING ubiquitin ligases. *Nat. Rev. Mol. Cell Biol.* 6 9–20 10.1038/nrm1547 15688063

[B59] Pickart C. M. Cohen R. E. (2004). Proteasomes and their kin: proteases in the machine age. *Nat. Rev. Mol. Cell Biol.* 5 177–187 10.1038/nrm1336 14990998

[B60] Rairdan G. J. Delaney T. P. (2002). Role of salicylic acid and NIM1/NPR1 in race-specific resistance in *Arabidopsis*. *Genetics* 161 803–811. 12072475 10.1093/genetics/161.2.803 PMC1462125

[B61] Rate D. N. Greenberg J. T. (2001). The *Arabidopsis* aberrant growth and death2 mutant shows resistance to Pseudomonas syringae and reveals a role for NPR1 in suppressing hypersensitive cell death. *Plant J.* 27 203–211 10.1046/j.0960-7412.2001.1075 11532166

[B62] Reid G. Hubner M. R. Metivier R. Brand H. Denger S. Manu D. (2003). Cyclic, proteasome-mediated turnover of unliganded and liganded ERα on responsive promoters is an integral feature of estrogen signaling. *Mol. Cell* 11 695–707 10.1016/S1097-2765(03)00090-X 12667452

[B63] Ryals J. Weymann K. Lawton K. Friedrich L. Ellis D. Steiner H. Y. (1997). The *Arabidopsis* NIM1 protein shows homology to the mammalian transcription factor inhibitor IkB. *Plant Cell* 9 425–439. 9090885 10.1105/tpc.9.3.425 PMC156928

[B64] Sadanandom A. Bailey M. Ewan R. Lee J. Nelis S. (2012). The ubiquitin-proteasome system: central modifier of plant signalling. *New Phytol.* 196 13–28 10.1111/j.1469-8137.2012.04266.x 22897362

[B65] Salghetti S. E. Caudy A. A. Chenoweth J. G. Tansey W. P. (2001). Regulation of transcriptional activation domain function by ubiquitin. *Science* 293 1651–1653 10.1126/science.1062079 11463878

[B66] Salghetti S. E. Muratani M. Wijnen H. Futcher B. Tansey W. P. (2000). Functional overlap of sequences that activate transcription and signal ubiquitin-mediated proteolysis. *Proc. Natl. Acad. Sci. U.S.A.* 97 3118–3123 10.1073/pnas.97.7.3118 10706616 PMC16202

[B67] Santner A. Estelle M. (2009). Recent advances and emerging trends in plant hormone signalling. *Nature* 459 1071–1078 10.1038/nature08122 19553990

[B68] Shah J. Tsui F. Klessig D. F. (1997). Characterization of a salicylic acid-insensitive mutant (sai1) of *Arabidopsis thaliana*, identified in a selective screen utilizing the SA-inducible expression of the tms2 gene. *Mol. Plant Microbe Interact.* 10 69–78 10.1094/MPMI.1997.10.1.69 9002272

[B69] Shi Z. Maximova S. Liu Y. Verica J. Guiltinan M. J. (2013). The salicylic acid receptor NPR3 is a negative regulator of the transcriptional defense response during early flower development in *Arabidopsis*. *Mol. Plant* 6 802–816 10.1093/mp/sss091 22986789

[B70] Shimono M. Koga H. Akagi A. Hayashi N. Goto S. Sawada M. (2012). Rice WRKY45 plays important roles in fungal and bacterial disease resistance. *Mol. Plant Pathol.* 13 83–94 10.1111/j.1364-3703.2011.00732.x 21726399 PMC6638719

[B71] Shimono M. Sugano S. Nakayama A. Jiang C. J. Ono K. Toki S. (2007). Rice WRKY45 plays a crucial role in benzothiadiazole-inducible blast resistance. *Plant Cell* 19 2064–2076 10.1105/tpc.106.046250 17601827 PMC1955718

[B72] Shirasu K. (2009). The HSP90-SGT1 chaperone complex for NLR immune sensors. *Annu. Rev. Plant Biol.* 60 139–164 10.1146/annurev.arplant.59.032607.092906 19014346

[B73] Smalle J. Vierstra R. D. (2004). The ubiquitin 26S proteasome proteolytic pathway. *Annu. Rev. Plant Biol.* 55 555–590 10.1146/annurev.arplant.55.031903.141801 15377232

[B74] Spoel S. H. Dong X. N. (2012). How do plants achieve immunity? defence without specialized immune cells. *Nat. Rev. Immunol.* 12 89–100 10.1038/nri3141 22273771

[B75] Spoel S. H. Mou Z. Tada Y. Spivey N. W. Genschik P. Dong X. (2009). Proteasome-mediated turnover of the transcription coactivator NPR1 plays dual roles in regulating plant immunity. *Cell* 137 860–872 10.1016/j.cell.2009.03.038 19490895 PMC2704463

[B76] Spoel S. H. Tada Y. Loake G. J. (2010). Post-translational protein modification as a tool for transcription reprogramming. *New Phytol.* 186 333–339 10.1111/j.1469-8137.2009.03125.x 20015068

[B77] Stogios P. J. Chen L. Prive G. G. (2007). Crystal structure of the BTB domain from the LRF/ZBTB7 transcriptional regulator. *Protein Sci.* 16 336–342 10.1110/ps.062660907 17189472 PMC2203294

[B78] Stokes T. L. Kunkel B. N. Richards E. J. (2002). Epigenetic variation in *Arabidopsis* disease resistance. *Genes Dev.* 16 171–182 10.1101/gad.952102 11799061 PMC155322

[B79] Sugano S. Jiang C. J. Miyazawa S. I. Masumoto C. Yazawa K. Hayashi N. (2010). Role of OsNPR1 in rice defense program as revealed by genome-wide expression analysis. *Plant Mol. Biol.* 74 549–562 10.1007/s11103-010-9695-3 20924648

[B80] Tada Y. Spoel S. H. Pajerowska-Mukhtar K. Mou Z. Song J. Wang C. (2008). Plant immunity requires conformational changes of NPR1 via S-nitrosylation and thioredoxins. *Science* 321 952–956 10.1126/science.1156970 18635760 PMC3833675

[B81] Tang L.-Y. Yamashita M. Coussens N. P. Tang Y. Wang X. Li C. (2011). Ablation of Smurf2 reveals an inhibition in TGF-β signalling through multiple mono-ubiquitination of Smad3. *EMBO J.* 30 4777–4789 10.1038/emboj.2011.393 22045334 PMC3243605

[B82] Thrower J. S. Hoffman L. Rechsteiner M. Pickart C. M. (2000). Recognition of the polyubiquitin proteolytic signal. *EMBO J.* 19 94–102 10.1093/emboj/19.1.94 10619848 PMC1171781

[B83] Trujillo M. Shirasu K. (2010). Ubiquitination in plant immunity. *Curr. Opin. Plant Biol.* 13 402–408 10.1016/j.pbi.2010.04.002 20471305

[B84] van den Burg H. A. Tsitsigiannis D. I. Rowland O. Lo J. Rallapalli G. Maclean D. (2008). The F-box protein ACRE189/ACIF1 regulates cell death and defense responses activated during pathogen recognition in tobacco and tomato. *Plant Cell* 20 697–719 10.1105/tpc.107.056978 18375657 PMC2329923

[B85] van der Horst A. de Vries-Smits A. M. M. Brenkman A. B. van Triest M. H. van den Broek N. Colland F. (2006). FOXO4 transcriptional activity is regulated by monoubiquitination and USP7/HAUSP. *Nat. Cell Biol.* 8 1064–1073 10.1038/ncb1469 16964248

[B86] van Ooijen G. Lukasik E. Van Den Burg H. A. Vossen J. H. Cornelissen B. J. C. Takken F. L. W. (2010). The small heat shock protein 20 RSI2 interacts with and is required for stability and function of tomato resistance protein I-2. *Plant J.* 63 563–572 10.1111/j.1365-313X.2010.04260.x 20497382 PMC2988412

[B87] van Ooijen G. van den Burg H. A. Cornelissen B. J. C. Takken F. L. W. (2007). Structure and function of resistance proteins in solanaceous plants. *Annu. Rev. Phytopathol.* 45 43–72 10.1146/annurev.phyto.45.062806.094430 17367271

[B88] Vierstra R. D. (2009). The ubiquitin-26S proteasome system at the nexus of plant biology. *Nat. Rev. Mol. Cell Biol.* 10 385–397 10.1038/nrm2688 19424292

[B89] Walsh C. K. Sadanandom A. (2014). Ubiquitin chain topology in plant cell signaling: a new facet to an evergreen story. *Front. Plant Sci.* 5:122 10.3389/fpls.2014.00122 PMC3978257 24744767

[B90] Wang D. Amornsiripanitch N. Dong X. (2006). A genomic approach to identify regulatory nodes in the transcriptional network of systemic acquired resistance in plants. *PLoS Pathog.* 2:e123 10.1371/journal.ppat.0020123 PMC1635530 17096590

[B91] Weigel R. R. Pfitzner U. M. Gatz C. (2005). Interaction of NIMIN1 with NPR1 modulates PR gene expression in *Arabidopsis*. *Plant Cell* 17 1279–1291 10.1105/tpc.104.027441 15749762 PMC1088002

[B92] Wu R. C. Feng Q. Lonard D. M. O’Malley B. W. (2007). SRC-3 coactivator functional lifetime is regulated by a phospho-dependent ubiquitin time clock. *Cell* 129 1125–1140 10.1016/j.cell.2007.04.039 17574025

[B93] Wu Y. Zhang D. Chu J. Y. Boyle P. Wang Y. Brindle I. D. (2012). The *Arabidopsis* NPR1 protein is a receptor for the plant defense hormone salicylic acid. *Cell Rep.* 1 639–647 10.1016/j.celrep.2012.05.008 22813739

[B94] Yi H. Richards E. J. (2009). Gene duplication and hypermutation of the pathogen resistance gene SNC1 in the *Arabidopsis* bal variant. *Genetics* 183 1227–1234 10.1534/genetics.109.105569 19797048 PMC2787416

[B95] Zhai Q. Yan L. Tan D. Chen R. Sun J. Gao L. (2013). Phosphorylation-coupled proteolysis of the transcription factor MYC2 is important for jasmonate-signaled plant immunity. *PLoS Genet.* 9:e1003422 10.1371/journal.pgen.1003422 PMC3616909 23593022

[B96] Zhang Y. Cheng Y. T. Qu N. Zhao Q. Bi D. Li X. (2006). Negative regulation of defense responses in *Arabidopsis* by two NPR1 paralogs. *Plant J.* 48 647–656 10.1111/j.1365-313X.2006.02903.x 17076807

[B97] Zhang Y. Fan W. Kinkema M. Li X. Dong X. (1999). Interaction of NPR1 with basic leucine zipper protein transcription factors that bind sequences required for salicylic acid induction of the PR-1 gene. *Proc. Natl. Acad. Sci. U.S.A.* 96 6523–6528 10.1073/pnas.96.11.6523 10339621 PMC26915

[B98] Zhang Y. Tessaro M. J. Lassner M. Li X. (2003). Knockout analysis of *Arabidopsis* transcription factors TGA2 TGA5 and TGA6 reveals their redundant and essential roles in systemic acquired resistance. *Plant Cell* 15 2647–2653 10.1105/tpc.014894 14576289 PMC280568

[B99] Zhou J. M. Trifa Y. Silva H. Pontier D. Lam E. Shah J. (2000). NPR1 differentially interacts with members of the TGA/OBF family of transcription factors that bind an element of the PR-1 gene required for induction by salicylic acid. *Mol. Plant Microbe Interact.* 13 191–202 10.1094/MPMI.2000.13.2.191 10659709

[B100] Zhou W. Slingerland J. M. (2014). Links between oestrogen receptor activation and proteolysis: relevance to hormone-regulated cancer therapy. *Nat. Rev. Cancer* 14 26–38 10.1038/nrc3622 24505618

[B101] Zhuang M. Calabrese M. F. Liu J. Waddell M. B. Nourse A. Hammel M. (2009). Structures of SPOP-substrate complexes: insights into molecular architectures of BTB-Cul3 ubiquitin ligases. *Mol. Cell* 36 39–50 10.1016/j.molcel.2009.09.022 19818708 PMC2847577

